# Dietary Oligosaccharides Alter Blood and Fecal Metabolites in Holstein Dairy Calves

**DOI:** 10.3390/ani16010016

**Published:** 2025-12-20

**Authors:** Rafaela Santos, Luciana M. Kluppel, Nirosh Senevirathne, Juliano Peres Prietsch, Venkateswarlu Sunkesula, Olufemi Akinkuotu, Babafela Awosile, Erminio Trevisi, Fernanda Rosa

**Affiliations:** 1 School of Veterinary Medicine, Texas Tech University, Amarillo, TX 79106, USA; rafsanto@ttu.edu (R.S.); luciana.kluppel@ttu.edu (L.M.K.); julianoprie@gmail.com (J.P.P.); olufemi.akinkuotu@ttu.edu (O.A.); babafela.awosile@ttu.edu (B.A.); 2 Department of Animal Sciences, Cornell University, Ithaca, NY 14850, USA; ns745@cornell.edu; 3 Department of Animal Sciences, Federal University of Pelotas, Pelotas 96010-610, Brazil; 4 Idaho Milk Products, Jerome, ID 83338, USA; vsunkesula@idahomilk.us; 5 Department of Animal Sciences, Food and Nutrition (DIANA), Faculty of Agriculture, Food and Environmental Science, Università Cattolica del Sacro Cuore, 20123 Milan, Italy; erminio.trevisi@unicatt.it; 6 Romeo and Enrica Invernizzi Research Center for Sustainable Dairy Production of the Università Cattolica del Sacro Cuore (CREI), 20123 Milan, Italy

**Keywords:** dairy calves, oligosaccharides, metabolites, preweaning, health

## Abstract

Neonatal and weaning periods are the most challenging phases for enteric infections in dairy calves. While different approaches are used to minimize calf health issues, the neonatal diet remains the core approach to enhance pre- and post-weaning animal health and performance. This pilot study aimed to evaluate blood biomarkers of inflammation and fecal metabolites in calves supplemented with oligosaccharide mixture (OS) during the preweaning stage. Sixteen newborn Holstein dairy calves (eight females and eight males) were randomly assigned into two groups: control with no supplementation, or treatment with 50 g/day of OS added to the pasteurized waste milk feeding. Both control and OS-treated calves were fed pasteurized waste milk during the preweaning period and weaned at seven weeks of age. Blood and fecal samples were collected weekly. Fecal fatty acid metabolites were downregulated in treated calves compared to control calves (*p* ≤ 0.05). Markers of inflammation (i.e., haptoglobin) or liver response (i.e., albumin, paraoxonase) did not differ between groups (*p* > 0.05). Overall, these findings suggest that dietary intervention with oligosaccharides can affect host metabolic pathways.

## Simple Summary

This study evaluated the effects of supplementing oligosaccharides (non-digestible carbohydrates) on health parameters of pre-weaned dairy calves. We found that oligosaccharide supplementation influenced immune markers and gut metabolism, suggesting improved inflammatory response and nutrient absorption. These findings highlight the potential of neonatal dietary interventions to enhance calf health during vulnerable early-life stages. The results from this pilot study may help guide more sustainable and health-focused practices in the dairy industry.

## 1. Introduction

Early-life nutrition plays a critical role in shaping the health, growth, and productivity of dairy calves. The preweaning period is a particularly vulnerable window in dairy calf management marked by rapid gastrointestinal development, immune system maturation, and a high risk of enteric disorders such as calf scour or diarrhea [1]. Nutritional interventions that support gut health and microbial balance during this phase are essential for improving calf performance and long-term outcomes in dairy herds [2].

Prebiotics or non-digestible oligosaccharides (non-digestible dietary carbohydrates), including galacto-oligosaccharides (GOS), fructo-oligosaccharides (FOS), and mannan-oligosaccharides (MOS), serve as substrates that are metabolized by the host microbial community. Non-digestible oligosaccharides increase the production of short-chain fatty acids (SCFAs), among other properties that overall improve growth and promote health in the host [2,3]. Dietary oligosaccharides resist hydrolysis by endogenous enzymes in the upper gastrointestinal tract and are selectively fermented by beneficial commensal bacteria, such as *Bifidobacterium* and *Lactobacillus*, in the colon [4,5,6]. Through this mechanism, oligosaccharide supplementation may enhance microbial diversity, improve intestinal barrier integrity, modulate immune responses, and reduce colonization by pathogenic bacteria.

Studies in neonatal calves have reported that dietary GOS and other prebiotic oligosaccharides can positively influence gut morphology, increase SCFA production, and decrease the incidence of diarrhea [7,8]. These changes are associated with improvements in nutrient absorption, feed efficiency, and overall growth performance [9,10]. Similarly, FOS supplementation has shown benefits in promoting hindgut fermentation, extending the persistence of *Bifidobacterium*, and increasing fecal concentrations of acetate and propionate, which were positively correlated with fecal SCFA levels and average daily gain (ADG) in preweaned calves [11]. MOS supplementation has also increased rumen papillae length and jejunal villi height in neonatal calves [3]. However, the specific effects may vary depending on the type, dose, and duration of oligosaccharide supplementation, as well as the basal diet and management conditions. Although some studies have shown growth and microbial colonization benefits from oligosaccharides supplementation, the effects of these compounds on immune and metabolic functions in neonatal calves warrant further investigation.

Given the increasing interest in antibiotic-free strategies for calf rearing, understanding the role of prebiotic oligosaccharides in modulating gastrointestinal health and growth in preweaning dairy calves is of both scientific and practical relevance. In this context, our pilot study aimed to evaluate the effects of dietary supplementation with oligosaccharides mixture on blood metabolic and inflammatory parameters, and on fecal metabolome as a proxy of gut microbial dynamics in neonatal dairy calves.

## 2. Materials and Methods

### 2.1. Experimental Design

The animal trial was conducted under protocols approved by the South Dakota State University (SDSU) Institutional Animal Care and Use Committee (Protocol # 17-034E). The calf performance data (e.g., body weight, health scores) and nutrient intake data from this study were previously published [9]. From the original cohort of calves, sixteen newborn Holstein calves (8 females and 8 males) were used in the present study. All calves were housed in individual calf hutches at the former SDSU Dairy Research and Training Facility (Brookings, SD, USA) with ad libitum access to water. Immediately after birth, calves were fed high-quality colostrum (3.79 L of colostrum with >50 mg of IgG/mL), followed by a second feeding of colostrum (2.83 L ~6 h after first feeding). Calves were weaned at 7 weeks of age.

### 2.2. Experimental Groups

After colostrum feeding, calves were randomly assigned to a control or OS-treatment group (*n* = 8/group [4 females and 4 males/group]) in a randomized complete block design in which animals were blocked by sex and birthdate. All calves received 2.83 L of pasteurized waste milk twice daily from weeks 1 to 5 of age and once daily during week 6, regardless of group. Control calves were fed pasteurized waste milk from week 1 to 6 of age without supplementation. OS-treated calves received pasteurized waste milk with 25 g of oligosaccharide mixture added to each morning and afternoon feeding from weeks 1 to 5 of age (total of 50 g of supplementation/day). At week 6, treated calves were fed 25 g of oligosaccharides mixture in the morning feeding, and another 25 g was added to the calf starter pellets.

In this study, the oligosaccharide supplementation dose of 50 g/day was selected based on a previous study that tested the effects of an OS-mixture containing FOS fed at rates of 30, 45, and 60 g/day to Holstein calves during the preweaning period [12]. Additionally, the dietary oligosaccharides mixture fed to the treatment group in our study was derived from milk permeate through advanced membrane filtration techniques developed by Idaho Milk Products (Jerome, ID, USA). This process concentrates the carbohydrate fraction of milk permeate, resulting in a dietary mixture including lactose (disaccharide) and enzymatically synthesized GOS that are soluble, serving as an effective carbohydrate source. Nutrient composition of pasteurized waste milk and of oligosaccharide mixture fed to calves in this study was previously reported by Senevirathne et al. [9].

### 2.3. Sample Collection

Blood samples were collected prior to morning feeding from the jugular vein using 20-gauge BD vacutainer needles (Becton Dickinson, Franklin Lakes, NJ, USA) and into vacuum tubes (10 mL, BD Vacutainer^®^, Franklin Lakes, NJ, USA) containing lithium heparin. Blood samples were collected at 24 h after birth and after colostrum ingestion (i.e., 0 d), and subsequent samples were taken at 7 and 21 days of age (±1). Following blood collection, tubes were placed on ice until centrifugation. Plasma samples were obtained by centrifugation of lithium heparin tubes at 1200× *g* for 15 min at 4 °C. Aliquots of plasma were frozen in a −80 °C freezer until further analysis. After morning feeding, fresh fecal samples (~10 g) were collected from each calf via rectum stimulation on two different time points (14 ± 1 and 42 ± 1 days of age). Fecal samples were collected into 2 mL cryogenic vials (Corning^®^, Cat# CLS431417, Corning, NY, USA) and immediately flash-frozen in liquid N2. Samples were kept at −80 °C until further metabolomics analysis.

### 2.4. Blood Metabolites and Statistical Analysis

Plasma samples were analyzed for glucose, total proteins, albumin, cholesterol, glutamic-oxaloacetic transaminase (GOT), gamma-glutamyl transferase (GGT), and alkaline phosphatase (ALP) using kits from Instrumentation Laboratory Spa (Werfen Co., Milan, Italy) in the ILAB 600 clinical auto-analyzer (Instrumentation Laboratory, Lexington, MA, USA). Globulin levels were calculated as the difference between total proteins and albumin. Total plasma reactive oxygen metabolites (ROM) were measured using commercial kits from Diacron International s.r.l. (Grosseto, Italy). Zinc and non-esterified fatty acids (NEFA) were analyzed using Wako Chemicals Europe GmbH (Neuss, Germany); β-hydroxybutyric acid (BHB) was analyzed using kits purchased from Randox Laboratories Ltd., Crumlin, UK. Haptoglobin was analyzed using the method described by Skinner et al. [13]. Analyses of ferric reducing antioxidant power (FRAP) were performed with the colorimetric method of Benzie and Strain [14]. The concentrations of paraoxonase (PON) and myeloperoxidase (MPO) were determined as previously described by Trevisi et al. [15].

All analyses were performed using the computing environment of R software (v 4.3.2). A total of 16 blood parameters were included as the explanatory variables with sex of the calves, time (day 0, day 7, and day 21 of age), and treatment (control vs. OS), as well as their interactions as the fixed effects. A calf nested within treatment was included as a random effect. Initial descriptive statistics were carried out to summarize the outcomes of interest by different explanatory variables. The data structure in the study is multivariate repeated measurements of 16 different blood parameters at three different times (day 0, day 7, and day 21), depending on the treatment groups. Multivariate post hoc comparison analysis was carried out for the statistically significant independent variables. Further repeated-measure univariate analysis of covariance (ANCOVA) was performed to determine which univariate blood parameter or combination of blood parameters contributed to the statistical significance of the global multivariate analysis. Following the multivariate analysis, we also performed repeated-measure ANCOVA for each of the outcomes, exploring the effects of the three independent variables. For each statistically significant variable, we performed post hoc analysis, adjusting for multiple comparisons using Bonferroni correction. For each repeated measure ANCOVA, the assumption of sphericity was explored using Mauchly’s test; whenever there was a violation of the sphericity assumption, Greenhouse–Geisser sphericity correction was applied to factors violating this assumption. Statistical significance and tendencies were considered at *p* ≤ 0.05 and 0.05 ≤ *p* ≤ 0.10, respectively.

### 2.5. Fecal Metabolite Profiling and Statistical Analysis

Fecal samples were submitted to the West Coast Metabolomics Center at the University of California, Davis for primary metabolomics analyses using gas chromatography/mass spectrometry (GC/MS, LECO, St. Joseph, MI, USA). Approximately 15 mg (±0.3 mg) of bovine stool from each calf was used to have a pool for quality control (QC) during the metabolome processing procedures. Detailed GC/MS instrument conditions and the process of the metabolome data were reported previously [16,17,18,19]. Compounds were identified by comparison with the Fiehn lab BinBase database annotations [17], database identifier (i.e., InChI key [20]), the compound annotation metadata (i.e., retention index, quantification mass, BinBase identifier, and mass spectrum), and PubChem annotation [21]. A list of peak heights, retention time, and mass-to-charge ratios (*m*/*z*) was obtained. The metabolome data were normalized by the average mTIC (mass-to-charge ratio representing the sum of all peak heights for all known metabolites) of the samples. A total of 553 metabolites were detected in all samples, including 187 annotated and 366 unknown metabolites. The unknown metabolites were excluded from the current analysis. The normalized mTIC metabolites were analyzed using an online free tool, MetaboAnalyst 6.0 (https://www.metaboanalyst.ca/, access date 6 May 2025) [22]. The metabolome data within MetaboAnalyst were analyzed following previous protocols [18,19]. Briefly, metabolites were normalized by the sum of all identified metabolites, autoscaled (mean-centered and divided by the standard deviation of each variable), and log-transformed prior to downstream statistical analysis [23]. Multivariate analysis was performed using supervised partial least-squares discriminant analysis (PLS-DA), followed by a *t*-test between groups at each time point (i.e., 14 and 42 days of age) and the calculation of the fold change (FC) for each metabolite. The heatmap was generated based on the Pearson distance measure and the Ward clustering algorithm using the top 20 metabolites selected by the PLS-DA analysis. Metabolites most strongly influencing discrimination between groups were selected based on the following criteria: variable importance in the projection (VIP) score > 1.0 [24,25] and statistical significance and tendencies, which were considered at *p* ≤ 0.05 and 0.05 ≤ *p* ≤ 0.10, respectively.

## 3. Results

Metabolites data, including the interaction effects, are presented in Table 1.

### 3.1. Energy Metabolism

There was a time effect (*p* < 0.01) observed for BHB, which was reflected by lower BHB concentration on day 7 compared to days 0 and 21 in the blood of all calves regardless of treatment. Blood NEFA tended to be lower (*p* = 0.10) in the OS-treated group compared to the control. However, blood glucose was not affected by treatment (*p* = 0.12), time (*p* = 0.56), sex (*p* = 0.83), or by their interaction (*p* = 0.47).

### 3.2. Liver Function Metabolites

The effect of time was observed for total proteins, globulins, and GGT (*p* ≤ 0.01), where all metabolites decreased in the blood of all calves over time, regardless of treatment. Similarly, GOT decreased in the blood of all calves over time (*p* < 0.01). However, blood albumin and cholesterol increased over time in all calves (*p* < 0.01).

### 3.3. Inflammatory and Oxidative Stress Parameters

A group × time × sex interaction effect (*p* = 0.02) was observed for the enzyme MPO, where OS-treated female calves had lower MPO activity in blood compared to female controls at 21 days of age. The effect of time was observed for PON (*p* < 0.01), by which its enzymatic activity increased in the blood of all calves over time, regardless of treatment. Blood PON levels were higher on day 21 (*p* < 0.01) and day 7 (*p* = 0.03) compared to day 0. Blood haptoglobin was not affected by treatment, time, sex, or interaction. There was a time effect (*p* < 0.01) on the FRAP levels that decreased over time regardless of treatment. Similarly, there was a time effect (*p* < 0.01) for blood ROM reflected by an increase over time irrespective of the treatment status of the calves. ROM levels were higher on day 21 and day 7 compared to day 0 (*p* < 0.05). Additionally, blood zinc level did not differ between treatment, time, sex, or interaction.

### 3.4. Fecal Metabolite Profile at 14 and 42 Days of Age

**Figure 1:**
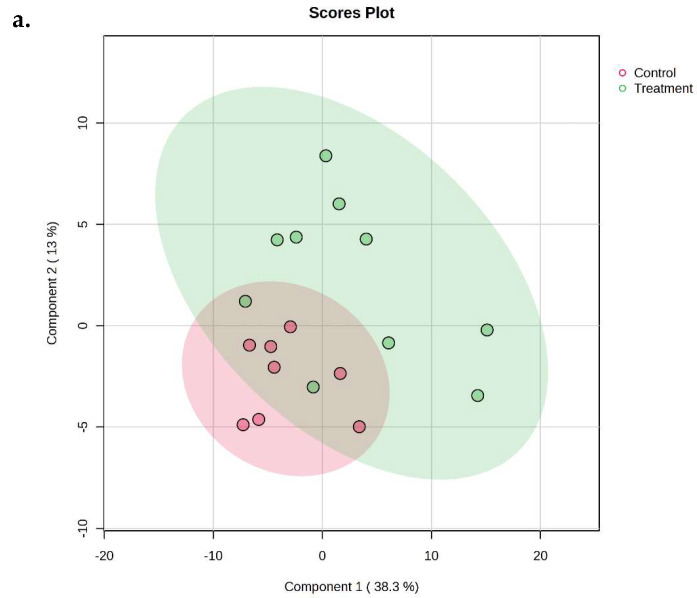

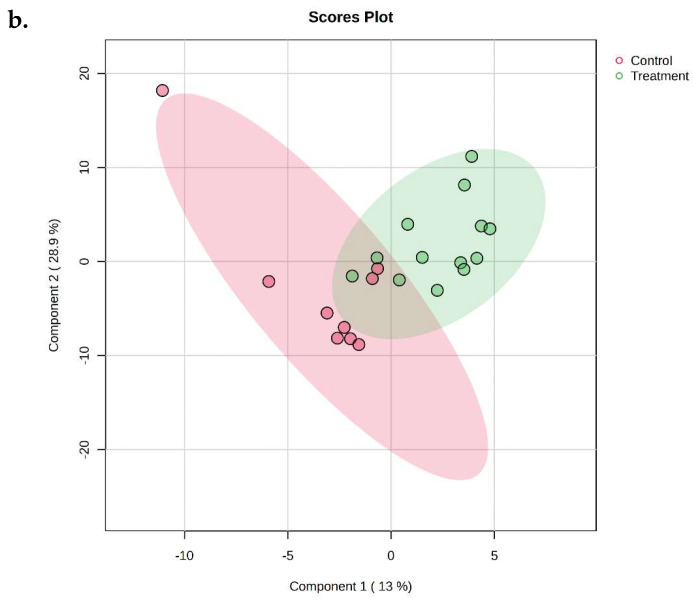
Two-dimensional scores plot of supervised partial least-squares discriminant analysis (PLS-DA) model showing all the annotated metabolites identified across the fecal samples of Holstein dairy calves fed pasteurized waste milk without supplementation (Control) or pasteurized waste milk supplemented with dietary oligosaccharides (Treatment). Panels depict fecal metabolites at 14 days of age (**a**) and at 42 days of age (**b**).

The full list of all detected metabolites in the feces of all calves (control and OS groups) at 14 and 42 days of age is presented in Supplementary Table S1. Although less apparent at 14 compared to 42 days of age, the supervised partial least-squares discriminant analysis (PLS-DA) using all the known (annotated) metabolites identified across the fecal samples demonstrated a separation of groups driven by variance in fecal metabolites at 14 and 42 days of age (Figure 1a and Figure 1b, respectively).

**Figure 2:**
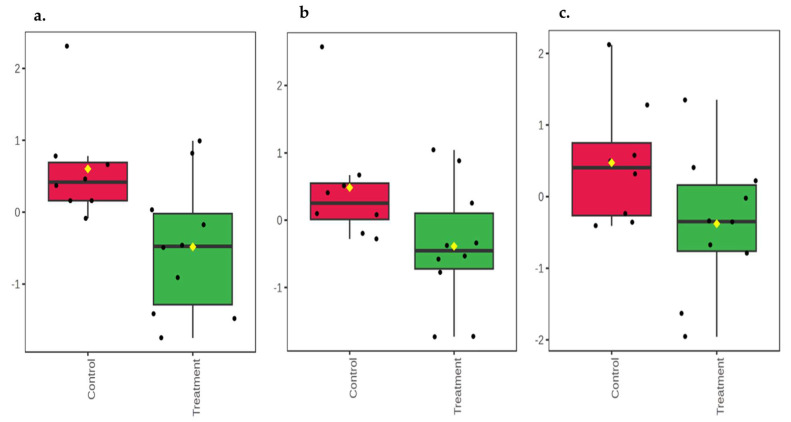
Boxplot of relative concentrations of altered metabolites in feces of Holstein dairy calves fed pasteurized waste milk without supplementation (Control) or pasteurized waste milk supplemented with dietary oligosaccharides (Treatment) at 14 days of age. Y-axes are represented as relative units. Data were normalized to the total spectral area. Due to this normalization process, we obtained a negative scale in the Y-axis (Metaboanalyst program analysis). The bar plots show the normalized values (mean +/− SD). Yellow rhombus represents the mean concentration of the metabolite within group. Medians are indicated by horizontal lines within each box, and single data points are indicated by circles. (**a**) urea (*p* = 0.02); (**b**) 2-ethylcaproic acid (*p* = 0.06); (**c**) palmitoleic acid (*p* = 0.07).

**Figure 3:**
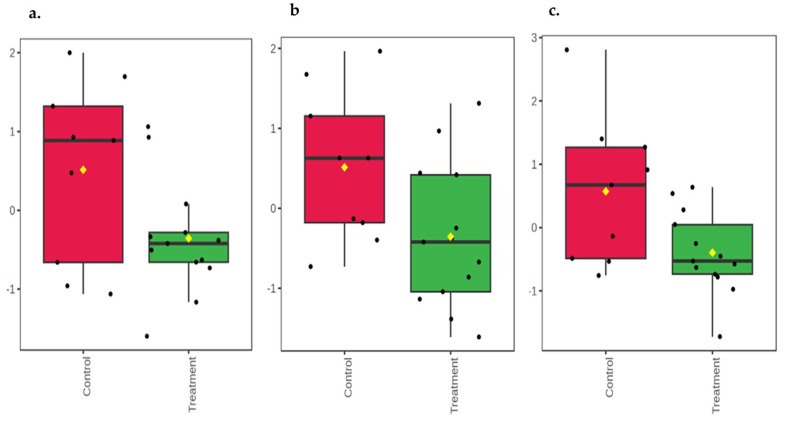
Boxplot of relative concentrations of altered metabolites in feces of Holstein dairy calves fed pasteurized waste milk without supplementation (Control) or pasteurized waste milk supplemented with dietary oligosaccharides (Treatment) at 42 days of age. Y-axes are represented as relative units. Data were normalized to the total spectral area. Due to this normalization process, we obtained a negative scale in the *Y*-axis (Metaboanalyst program analysis). The bar plots show the normalized values (mean +/− SD). Yellow rhombus represents the mean concentration of the metabolite within group. Medians are indicated by horizontal lines within each box, and single data points are indicated by circles. (**a**) 6-deoxyglucose (*p* = 0.04); (**b**) glucose (*p* = 0.04); (**c**) N-carbamoylaspartic acid (*p* = 0.02).

**Figure 4:**
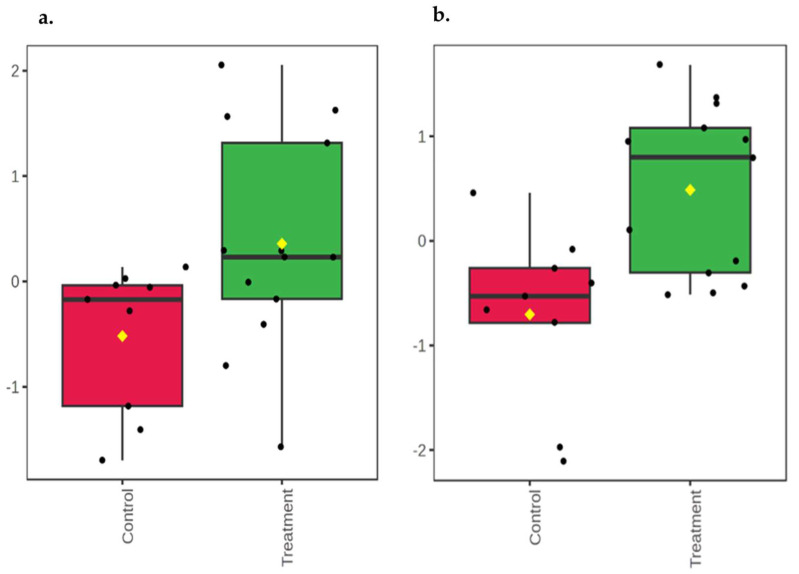
Boxplot of relative concentrations of altered metabolites in feces of Holstein dairy calves fed pasteurized waste milk without supplementation (Control) or pasteurized waste milk supplemented with dietary oligosaccharides (Treatment) at 42 days of age. Y-axes are represented as relative units. Data were normalized to the total spectral area. Due to this normalization process, we obtained a negative scale in the *Y*-axis (Metaboanalyst program analysis). The bar plots show the normalized values (mean +/− SD). Yellow rhombus represents the mean concentration of the metabolite within group. Medians are indicated by horizontal lines within each box, and single data points are indicated by circles. (**a**) aspartic acid (*p* = 0.04); (**b**) phenylalanine (*p* < 0.01).

**Figure 5:**
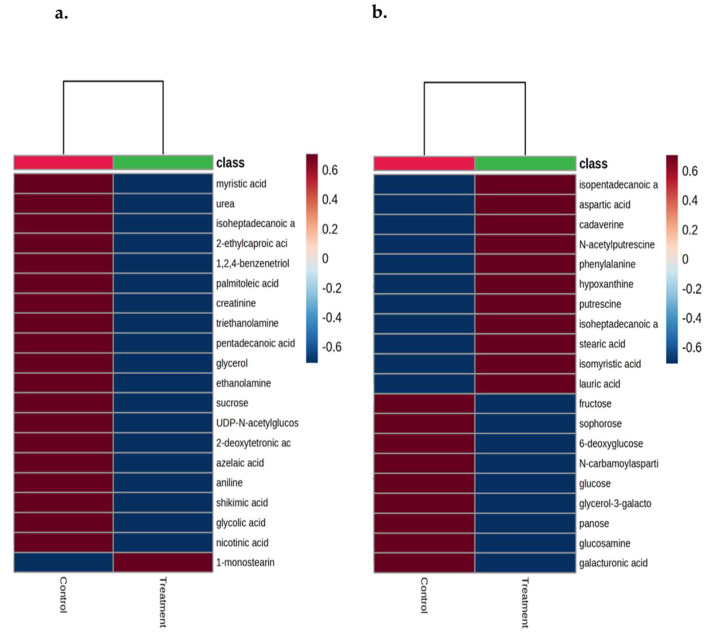
Heatmap generated using MetaboAnalyst (v. 6.0) highlighting the differential responses of the top 20 metabolites in the feces of Holstein dairy calves fed pasteurized waste milk without supplementation (Control) or pasteurized waste milk supplemented with dietary oligosaccharides (Treatment) at 14 days of age (**a**) and at 42 days of age (**b**). Peak area data scaled and plotted on a divergent scale. Relative abundance is shown with high abundance in red and low abundance in dark blue. The heatmap was generated based on the Pearson distance measure and the Ward clustering algorithm using the top 20 metabolites selected by the PLS-DA analysis. Metabolites most strongly influencing discrimination between groups were selected based on the variable importance in the projection (VIP) score > 1.0.

At 14 days of age, urea (*p* = 0.02; Figure 2a) was higher in the feces of the control group compared to the OS-treated group. 2-ethylcaproic acid (*p* = 0.06) and palmitoleic acid (*p* = 0.07) had a trend to be higher in the feces of the control calves compared to the OS-treated calves (Figure 2b and Figure 2c, respectively). At 42 days of age, 6-deoxyglucose, glucose, and N-carbamoylaspartic acid (*p* ≤ 0.04) were higher in the feces of the control calves compared to the OS-treated calves (Figure 3a and Figure 3b, and Figure 3c, respectively). The fecal metabolites aspartic acid (*p* = 0.04) and phenylalanine (*p* < 0.01) were higher in the feces of the OS-treated calves compared to the control calves (Figure 4a and Figure 4b, respectively). By contrast, there was a significant difference between the control and OS-treated group metabolite profile (based on the top 20 metabolites identified by VIP > 1.0) as demonstrated in the heatmap at 14 and 42 days of age (Figure 5a and Figure 5b, respectively). A list describing the categories (class and subclass) for each metabolite up- or down-regulated in the feces of all calves at 14 and 42 days of age is presented in Supplementary Table S2 and Supplementary Table S3, respectively.

## 4. Discussion

Calf health is critical to the adult cow’s performance and productivity, which also affects farm sustainability. Neonatal calves face challenges immediately after birth; not only do neonatal calves have to adapt to an extrauterine lifestyle, but they are also naturally challenged by environmental stressors, including microbial exposure and colonization. Such early-life stressors can cause calf scours, digestive disorders, and inhibit intake and growth [1]. In this scenario, early dietary interventions that can enhance calf health by improving the response to inflammation and by microbial modulation during the first 2–3 months of life are essential. In this study, we explored the effects of a dietary supplementation containing lactose and galacto-oligosaccharides (treatment) compared to a control diet (basal calf diet without the supplementation) on blood and fecal metabolites profile during the preweaning period of Holstein calves.

In neonatal calves, BHB levels increase in response to the microbial fermentation of dietary substrates in the rumen rather than body fat mobilization [26]. The lower concentrations of BHB at 7 days relative to 21 days of age were likely due to the different stimulation of the rumen fermentation, which is associated with increased crude protein and starch (e.g., starter pellets) [9]. Additionally, our findings are comparable to those observed by others [27,28,29], where BHB in neonatal calves increased as the calf aged, regardless of dietary treatments. This is also coherent with the trend for lower plasma NEFA in treated calves, which could suggest a lower mobilization of reserves during the first days of life.

During organ development and physiological adaptations of neonatal calves, proteins and lipoproteins synthesized by the liver can be affected by the maturation of the organ itself [30]. In turn, liver development and function can be affected by dietary compounds present in colostrum, including fatty acids, cholesterol, and growth factors that act as signaling molecules regulating metabolic pathways [31]. Therefore, blood metabolites in neonatal animals can be very difficult to interpret, especially if such a metabolite is a biomarker for disorders/diseases in adult animals. However, numerous studies have demonstrated that during an inflammatory state, the liver metabolism is altered in response to inflammation, resulting in increased production and release of positive acute phase proteins (e.g., haptoglobin) along with a decreased release of negative acute phase proteins (e.g., albumin, PON, cholesterol) [32,33,34]. In turn, lower albumin, cholesterol, and PON in the blood of animals have been associated with liver dysfunction or impairment [35]. In contrast, elevated liver enzymes in blood, including GGT, GOT, and ALP, are associated with liver cell damage or hepatic dysfunction [32]. In our study, regardless of diet, the increase in cholesterol and PON levels in the blood of all calves, along with the decrease in GGT and GOT from birth until 21 days of age indicate a positive liver maturation and function in those growing calves, which is in accordance with previous reports [27,36,37]. Although the higher levels of GGT, ALP, and GOT (to some extent) immediately after birth (time 0) can be attributed to the ingestion of colostrum [38,39]. Regarding inflammation, circulating MPO is indicative of phagocytosis by neutrophils, resulting in a local or systemic inflammatory responses [40]. Lower MPO in the female calves from the treatment group can be associated with less microbial infiltration in the gut. The latter suggests that oligosaccharides from the diet stimulate the growth of commensal microbiota on the host, which serve as decoy receptors to pathogenic microorganisms (i.e., blocking microbial infiltration) [41]. One plausible mechanism could be that MPO is a required enzyme in the NET formation employed by neutrophils [42]. Hence, higher MPO enzymatic activity can be expected when the body is challenged with an acute bacterial infection or fungal infection, which is often used as a biomarker for acute infections in humans [43,44].

Although most of the blood metabolites in this study only had a time effect (dependent on calf age), it is plausible that the small sample size used in our subsets (*n* = 8/group) could have inhibited some of the differences between groups. In addition to this, the calves enrolled in the treatment group received a fixed amount of 50 g/day regardless of the feeding strategy (i.e., mix in the waste milk or top-dressed in the starter pellets) or dry matter intake. Previous reports from this experimental cohort have shown that the treatment group showed increased body weight and feed intake as well as higher plasma BHB concentrations at the postweaning period [9], suggesting that the galacto-oligosaccharides fed to the treatment group enhanced calf performance during the postweaning relative to the preweaning stage. It is important to clarify that in the original animal experiment from this cohort study, calves were fed the treatment diet on the starter pellets during the postweaning period (total of 50 g/day top dressed) from week 7 to 12 of age.

Galacto-oligosaccharides have been used in human diets, especially in infant formulas, as an alternative to human milk oligosaccharides (naturally present in human milk). Human studies have shown that GOS-supplemented formula-fed infants (0.4 g/100 mL) had a higher abundance of Bifidobacterium and Lactobacillus genera compared to formula-fed infants without GOS supplementation [45]. Similarly, studies in monogastric animals and in ruminants have demonstrated that GOS supplementation promotes the growth of beneficial bacteria on the host, including Bifidobacterium and Lactobacillus, while preventing the colonization of pathogenic bacteria such as Clostridium perfringens [46,47,48,49]. Although GOS is used as a prebiotic in calf diets, there are limited studies that evaluated GOS supplementation on calf performance and, in particular, regarding the effects of GOS on health, including blood and fecal parameters [8,41,50]. As reviewed by Cangiano et al., several calf trials explored the benefits of MOS, which were commonly used in monogastric and ruminant diets, while fewer studies investigated GOS on calf health outcomes [2]. For instance, Castro et al. reported that preweaned calves fed milk replacer supplemented with GOS (3.4% of dry matter) had greater intestinal epithelial development compared to control calves; however, blood metabolites were not evaluated in the study [49]. Similarly, Quigley et al. reported a lower incidence of calf scours in pre-weaned calves fed milk replacer supplemented with galactosyl-lactose (1% of dry matter); however, blood inflammatory or metabolic parameters were not evaluated in this study [51]. Along with these fundings, Yu et al. fed milk supplemented with 2.5, 5, or 10 g/day/calf of GOS to newborn Holstein calves for 28 days. They reported that calves receiving 5 g/day of GOS experienced better growth performance, enhanced immune function, and nutrient utilization [8]. Another study using Holstein bull calves fed different levels (2, 4, or 8 g/calf/day) of GOS to the milk replacer from birth to 42 days of age demonstrated that 4 g/d of GOS supplementation to a milk replacer improved growth performance in neonatal calves [50]. Altogether, these studies could suggest that the benefits of oligosaccharide supplementation to young calves may be dependent on the amount fed to calves, calf age, and potential outcomes (growth vs. intestinal health). Importantly, different approaches to synthesizing oligosaccharides may provide enhanced prebiotic activity [52]. Hence, further studies are needed to explore different levels of di- and oligosaccharides, including their combination in early life, that can potentially enhance inflammatory responses and overall health of pre-weaned calves.

In the current study, most differential metabolites downregulated in the feces of 14-day-old Holstein dairy calves were fatty acyls, suggesting that oligosaccharides supplementation improved gut development, enhanced fat digestion/absorption, and favorable changes in fecal fatty acid profiles [53]. Oligosaccharides, including GOS, can serve as substrates to beneficial intestinal microorganisms that metabolize OS to short-chain fatty acids. Previous studies have shown that pre-weaned calves fed prebiotics had longer villi in the small intestine [49], which can potentially be regulated by higher levels of short-chain fatty acids in the gut [54,55]. The latter promotes intestinal development, including maturation of enterocytes (absorptive cells), thereby upregulating nutrient transporters and enzymes for host growth [8,56]. Thus, less fat is lost in feces because more is being utilized efficiently by the calf. Along with this, the downregulation of carbohydrate metabolites observed at 42 days old (six weeks) in the feces of calves supplemented with OS in our study can be an indicator of enhanced carbohydrate digestion and absorption in the gastrointestinal tract of OS-fed calves. Previous reports have demonstrated the link between the catabolism of short-chain fatty acids derived from carbohydrate digestion in the intestine [10,56], highlighting that improved fermentation both indicates more effective carbohydrate metabolism and less carbohydrate excretion.

In our study, higher levels of amino acids, including aspartic acid and phenylalanine, in the feces of OS-treated calves may indicate altered microbial metabolism [57]. Oligosaccharides selectively promote the growth of commensal bacteria, which shifts microbial fermentation toward greater incorporation of dietary nitrogen into microbial biomass [58]. In addition, oligosaccharides enhance mucosal barrier maturation and epithelial turnover, processes that naturally release endogenous proteins and peptides into the gut lumen. During periods of rapid epithelial remodeling, these endogenous substrates may contribute to higher concentrations of amino acids recovered in feces [59].

However, before further conclusions, some caveats must be made. In the present study, intestinal contents were not evaluated for short-chain fatty acids, nor were any other measurements taken. Overall, we speculate that the dietary oligosaccharides in this trial may have led to an increased flux of substrates into the hind gut, which provides a prebiotic effect. Furthermore, a limitation of this study was the small sample size since this was an exploratory-pilot study using a subset of animals from the original experiment that had been previously published [9]. In addition, optimization of enzymatic processes to obtain different oligosaccharides, as well as feeding rates, warrants further investigation to determine calf health responses while promoting calf growth.

## 5. Conclusions

The present study demonstrated that feeding a mixture containing lactose and galacto-oligosaccharides significantly reduces fecal carbohydrate and fatty acyl excretion in neonatal Holstein calves. OS-treated calves tended to have lower NEFA concentrations in blood compared to control calves. In summary, oligosaccharide supplementation can contribute to the modulation of host metabolism in neonatal Holstein calves.

## Figures and Tables

**Table 1:** Blood immunometabolic parameters during the neonatal period (from birth to 21 days of age) in Holstein dairy calves fed pasteurized waste milk without supplementation (Control) or pasteurized waste milk supplemented with dietary oligosaccharides (OS).

	Treatment ^1^			*p*-Value ^5^			
	Control ^2^	OS ^3^	SEM ^4^	Treatment	Time	Sex	T × T × S
**Energy metabolism**							
Glucose (mmol/L)	6.73	6.51	0.13	0.12	0.56	0.83	0.47
NEFA ^6^ (mmol/L)	0.30	0.25	0.02	0.10	0.17	0.42	0.37
BHB ^7^ (mmol/L)	0.083	0.081	0.01	0.46	<0.01	0.04	0.14
**Liver function**							
Albumin (g/L)	30.59	30.18	0.50	0.37	<0.01	0.16	0.59
Cholesterol (mmol/L)	2.20	2.45	0.22	0.38	<0.01	0.45	0.70
Total proteins (g/L)	67.66	67.75	1.61	0.85	0.01	0.76	0.07
Globulins (g/L)	37.07	37.57	1.55	0.98	<0.01	0.98	0.08
GGT ^8^ (U/L)	538.31	566.20	113.92	0.89	<0.01	0.99	0.64
ALP ^9^ (U/L)	483.27	483.89	54.00	0.60	0.31	0.93	0.89
GOT ^10^ (U/L)	67.09	70.34	7.08	0.94	<0.01	0.47	0.44
**Inflammation**							
Haptoglobin (g/L)	0.32	0.35	0.06	0.64	0.72	1.00	0.29
PON ^11^ (U/mL)	29.04	30.06	4.11	0.97	<0.01	0.33	0.60
MPO ^12^ (U/L)	392.61	407.51	19.75	0.66	0.12	0.04	0.02
**Oxidative Stress**							
ROM ^13^ (mg H_2_O_2_/100 mL)	13.07	13.69	0.73	0.69	<0.01	0.75	0.34
FRAP ^14^ (µmol/L)	149.60	142.26	7.61	0.33	<0.01	0.81	0.42
**Minerals**							
Zinc (µmol/L)	15.61	16.08	1.26	0.78	0.27	0.01	0.12

^1^ Group mean for each metabolite is presented. ^2^ Control—pasteurized waste milk without supplementation; *n* = 8 Holstein calves (4 males and 4 females). ^3^ OS—pasteurized waste milk + 50 g/calf/d of oligosaccharides mixture (OS); *n* = 8 Holstein calves (4 males and 4 females). ^4^ Largest standard error of the mean is shown. ^5^ *p*-value for treatment, sex, time (0, 7, 21 d), or interaction (Treatment [T] × Time [T] × Sex [S]). ^6^ NEFA = non-esterified fatty acids, ^7^ BHB = β-hydroxybutyric acid, ^8^ GGT = gamma-glutamyl transferase, ^9^ ALP = alkaline phosphatase, ^10^ GOT = glutamic-oxaloacetic transaminase, ^11^ PON = paraoxonase, ^12^ MPO = myeloperoxidase, ^13^ ROM = reactive oxygen metabolites, ^14^ FRAP = ferric reducing antioxidant power.

## Data Availability

The original contributions presented in this study are included in the article/Supplementary Material. Further inquiries can be directed to the corresponding author.

## Supplementary Materials

The following supporting information can be downloaded at https://www.mdpi.com/article/10.3390/ani16010016/s1: Table S1: All detected metabolites in the feces of control and treated calves at 14 and 42 days of age. Table S2: Classification of metabolites that were up- or down-regulated in the feces of all calves at 14 days of age. Table S3: Classification of metabolites that were up- or down-regulated in the feces of all calves at 42 days of age.

## Abbreviations

The following abbreviations are used in this manuscript:

SD	Standard deviation
OS	Oligosaccharides
