# Supplementary material for: Dietary Oligosaccharides Alter Blood and Fecal Metabolites in Holstein Dairy Calves
Source: Animals (Basel). 2025 Dec 20;16(1):16. doi: 10.3390/ani16010016 (PMC12785019; doi:10.3390/ani16010016)
Supplement: Supplementary file 1 [file animals-16-00016-s001.zip › Supplemental Tables S2-3.pdf]

Supplemental Tables S2-3

Supplemental Table S2. List of class and sub-class categories of the top 20 metabolites detected in the feces of Holstein dairy calves fed pasteurized waste milk without supplementation (Control) or pasteurized waste milk supplemented with dietary oligosaccharides (Treatment) at 14 days of age.

**Metabolite**	**Class**	**Sub Class**	**Source**
myristic acid	Fatty Acyls	Fatty acids and conjugates	https://www.hmdb.ca/metabolites
urea	Organic carbonic acids and derivatives	Ureas	https://www.hmdb.ca/metabolites
isoheptadecanoic acid	Fatty Acyls	Fatty acids and conjugates	https://www.hmdb.ca/metabolites
2-ethylcaproic acid	Fatty Acyls	Fatty acids and conjugates	https://www.hmdb.ca/metabolites
1,2,4-benzenetriol	Phenols	Benzenetriols and derivatives	https://www.hmdb.ca/metabolites
palmitoleic acid	Fatty Acyls	Fatty acids and conjugates	https://www.hmdb.ca/metabolites
creatinine	Carboxylic acids and derivatives	Amino acids, peptides, and analogues	https://www.hmdb.ca/metabolites
triethanolamine	Organonitrogen compounds	Amines	https://www.hmdb.ca/metabolites
pentadecanoic acid	Fatty Acyls	Fatty acids and conjugates	https://www.hmdb.ca/metabolites
glycerol	Organooxygen compounds	Carbohydrates and carbohydrate conjugates	https://www.hmdb.ca/metabolites
ethanolamine	Organonitrogen compounds	Amines	https://www.hmdb.ca/metabolites
sucrose	Organooxygen compounds	Carbohydrates and carbohydrate conjugates	https://www.hmdb.ca/metabolites
UDP-N-acetylglucos	Pyrimidine nucleotides	Pyrimidine nucleotide sugars	https://www.hmdb.ca/metabolites
2-deoxytetronic acid	Hydroxy acids and derivatives	Beta hydroxy acids and derivatives	https://www.hmdb.ca/metabolites
azelaic acid	Fatty Acyls	Fatty acids and conjugates	https://www.hmdb.ca/metabolites
aniline	Benzene and substituted derivatives	Aniline and substituted anilines	https://www.hmdb.ca/metabolites
shikimic acid	Organooxygen compounds	Alcohols and polyols	https://www.hmdb.ca/metabolites
glycolic acid	Hydroxy acids and derivatives	Alpha hydroxy acids and derivatives	https://www.hmdb.ca/metabolites
nicotinic acid	Pyridines and derivatives	Pyridinecarboxylic acids and derivatives	https://www.hmdb.ca/metabolites
1-monostearin	Glycerolipids	Monoradylglycerols	https://www.hmdb.ca/metabolites

Supplemental Table S3. List of class and sub-class categories of the top 20 metabolites detected in the feces of Holstein dairy calves fed pasteurized waste milk without supplementation (Control) or pasteurized waste milk supplemented with dietary oligosaccharides (Treatment) at 42 days of age.

**Metabolite**	**Class**	**Sub Class**	**Source**
isopentadecanoic acid	Glycerophospholipids	CDP-glycerols	https://www.hmdb.ca/metabolites
aspartic acid	Carboxylic acids and derivatives	Amino acids, peptides, and analogues	https://www.hmdb.ca/metabolites
cadaverine	Organonitrogen compounds	Amines	https://www.hmdb.ca/metabolites
N-acetylputrescine	Carboximidic acids and derivatives	Carboximidic acids	https://www.hmdb.ca/metabolites
phenylalanine	Carboxylic acids and derivatives	Amino acids, peptides, and analogues	https://www.hmdb.ca/metabolites
hypoxanthine	Imidazopyrimidines	Purines and purine derivatives	https://www.hmdb.ca/metabolites
putrescine	Organonitrogen compounds	Amines	https://www.hmdb.ca/metabolites
isoheptadecanoic a	Fatty Acyls	Fatty acids and conjugates	https://www.hmdb.ca/metabolites
stearic acid	Fatty Acyls	Fatty acids and conjugates	https://www.hmdb.ca/metabolites
isomyristic acid	Fatty Acyls	Fatty acids and conjugates	https://www.hmdb.ca/metabolites
lauric acid	Fatty Acyls	Fatty acids and conjugates	https://www.hmdb.ca/metabolites
fructose	Organooxygen compounds	Carbohydrates and carbohydrate conjugates	https://www.hmdb.ca/metabolites
sophorose	Organooxygen compounds	Carbohydrates and carbohydrate conjugates	https://www.mcdb.ca/metabolites/BMDB0062104
6-deoxyglucose	Organooxygen compounds	Carbohydrates and carbohydrate conjugates	https://www.hmdb.ca/metabolites/HMDB0000849
N-carbamoylasparti	Carboxylic acids and derivatives	Amino acids, peptides, and analogues	https://www.hmdb.ca/metabolites
glucose	Organooxygen compounds	Carbohydrates and carbohydrate conjugates	https://www.hmdb.ca/metabolites
glycerol-3-galacto	Glycerolipids	Glycosylglycerols	https://www.hmdb.ca/metabolites/HMDB0006790
panose	Organooxygen compounds	Carbohydrates and carbohydrate conjugates	https://www.hmdb.ca/metabolites
glucosamine	Organooxygen compounds	Carbohydrates and carbohydrate conjugates	https://www.hmdb.ca/metabolites
galacturonic acid	Organooxygen compounds	Carbohydrates and carbohydrate conjugates	https://www.hmdb.ca/metabolites
